# The Role of Rayleigh-Wood Anomalies and Surface Plasmons in Optical Enhancement for Nano-Gratings

**DOI:** 10.3390/nano8100809

**Published:** 2018-10-09

**Authors:** Ahmad A. Darweesh, Stephen J. Bauman, Desalegn T. Debu, Joseph B. Herzog

**Affiliations:** 1 Microelectronics-Photonics Graduate Program, 731 W. Dickson St., University of Arkansas, Fayetteville, Arkansas, AR 72701, USA; aadarwee@email.uark.edu (A.A.D.); sjbauman@email.uark.edu (S.J.B.); 2 Department of Physics, College of Science, AL-Nahrain University, Baghdad 10072, Iraq; 3 Department of Physics, 825 W. Dickson St., University of Arkansas, Fayetteville, Arkansas, AR 72701, USA; dtdebu@email.uark.edu; 4 R.B. Annis School of Engineering, University of Indianapolis, Indianapolis, IN 46227, USA

**Keywords:** plasmonics, nano-optics, Rayleigh-Wood anomalies, photo-sensing

## Abstract

We propose and report on the design of a 1-D metallo-dielectric nano-grating on a GaAs substrate. We numerically study the impact of grating period, slit and wire widths, and irradiating angle of incidence on the optical response. The optimal wire width, *w* = 160 nm, was chosen based on previous results from investigations into the influence of wire width and nano-slit dimensions on optical and electrical enhancements in metal-semiconductor-metal photodetectors. In this present project, resonant absorption and reflection modes were observed while varying the wire and nano-slit widths to study the unique optical modes generated by Rayleigh-Wood anomalies and surface plasmon polaritons. We observed sharp and diffuse changes in optical response to these anomalies, which may potentially be useful in applications such as photo-sensing and photodetectors. Additionally, we found that varying the slit width produced sharper, more intense anomalies in the optical spectrum than varying the wire width.

## 1. Introduction

When an incident light wave impinges on a plasmonic nanostructure, different types of resonance modes appear due to the fluctuation of free electrons in the conduction band of the metal. These modes are sensitive to several parameters, such as the energy, polarization and angle of the incident light, the nanostructure dimensions, and the refractive indices of the surrounding media. These oscillating modes are called “plasmons”, which are evanescent electromagnetic waves. The nature of these modes can determine the strength of the interaction between the incident light and the nanostructures; therefore, the optical response of plasmonic devices can be controlled if there is some degree of control over the incident light and/or nanostructures. Multiple light transmission mechanisms may be present and two types of anomalies, namely sharp and diffuse, were noticed by Wood in 1902 when experimenting with metallic diffraction gratings [1]. Such anomalies represent abrupt dips and peaks that appear in the spectrum as a function of wavelength or period at a fixed angle of incidence. The peaks, termed “Rayleigh’s anomaly”, occur due to “the passing-off of a spectrum of higher order” [2,3,4]. In other words, at a certain wavelength, a diffracted wave arises and propagates tangentially to the surface of the grating. This wavelength for a given grating is called “Rayleigh’s wavelength”. Somewhat surprisingly, such anomalies do not depend on the material of the nanostructure, but instead on the grating period, the incidence of the wavelength, and the refractive indices of the surrounding media. The dips are called “Wood’s anomaly” or “surface wave anomaly”; they emerge from the excitation of surface plasmons polaritons (SPPs) supported by the periodicity of the metallic nanostructure [5,6].

The aim of this research is to optimize a metallic nano-grating structure for wavelength-specific detection and sensing, and to study the impact of Rayleigh-Wood anomalies and surface plasmon polaritons on optical enhancement. In general, many researchers use a periodic structure with known dimensions, such as can be fabricated in a lab, for instance, and study the response over the relevant optical spectrum. However, for this paper we took the opposite approach; we focused on the desired wavelength and swept the structure dimensions and the incident angle. Several applications, such as optical filters [7,8], optical communications [9,10], surface-enhanced Raman spectroscopy (SERS) [11], photodetectors [12,13,14,15,16], and biosensors [17,18,19,20,21,22,23] could utilize these anomalies and surface plasmon phenomena to improve their performance. This study could provide dual benefits, depending on the application. For example, in photodetection enhancing the electric field in the substrate is the desired goal, and this enhancement can increase the electron-hole pair production, enhancing the photocurrent. Other applications based on scattered light focus on enhancement in the region of the incident medium. Because of this, it is important to enhance the optical response in the desired region, and this requires careful study of the modes and anomalies for the desired optical conditions.

## 2. Materials and Methods

**Figure 1:**
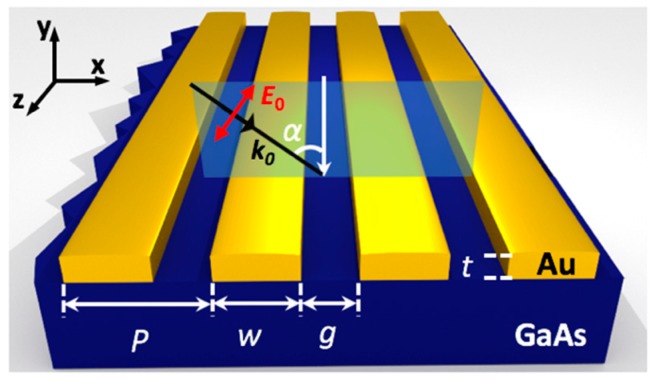
3-D schematic diagram of the modeled 1-D nano-grating structure consisting of gold nanowires on a GaAs substrate. *t* is the thickness of the wires and *w* is the wire width separated by nano-slits of width *g*. *P* = *w* + *g* is the structure period. Transverse-magnetic (TM) polarized light of wavelength *λ*_0_ = 875 nm is incident with an angle (α) from the top of the structure.

The general geometry that we consider in this paper is shown in Figure 1. The device consists of a substrate and a metallic grating with a thickness of *t* = 15 nm, corrugated by nano-slits of width *g* and nanowires of width *w*. A finite element method (FEM) was employed for modeling this system [24]. The simulated structure consisted of a single period (*P* = *w* + *g*), and periodic boundary conditions were set on either side in the x-direction (as labeled in Figure 1) to simulate an infinite array. The nanostructures were immersed in a superstrate with the material properties of air and laid on a substrate with the properties of GaAs. A plane wave with wavenumber *k*_0_ was incident on the nanostructures from the top with angle *α* and was transverse-magnetic (TM) polarized (parallel to the incident plane). The operating wavelength, *λ*_0_, was fixed at 875 nm, near the bandgap of GaAs, so that we could optimize GaAs photodetectors. The top edges of the nanostructures were rounded with a 3-nm radius to more closely match the fabricated structures, as demonstrated by standard lithographic methods [25,26]. Optical properties of materials (frequency-dependent) were taken from experimental results [27,28].

The general equation of a diffraction grating is given by Equation (1):

(1) $$\;n_{sub}\sin\left(\beta_{m}\right)=\;n_{sup}\sin\left(\alpha_{inc}\right)\;\pm m\lambda_{0}/P\;$$

where *m* is an integer referring to the order of the diffracted wave, *n*_sub_ and *β*_m_ are the refractive index and diffraction angle in the transmitted medium (substrate), and *n*_sup_ and *α*_inc_ are the refractive index and light angle in the incident medium (superstrate), respectively [29,30]. Since we used *λ*_0_ = 875 nm, which is located in the infrared (IR) region and equal to the bandgap energy of GaAs, it was important to state the substrate and superstrate indices of refraction at the chosen wavelength: *n*_sub_ = 3.62 and *n*_sup_ = 1. Rayleigh’s anomaly manifests when *β*_m_ = 90° because the diffracted wave propagates parallel to the grating, causing a drop in the transmission of light through the grating into the substrate medium. In this situation, the grating equation at normal incidence becomes:

(2) $$\;P=\;m\lambda_{0}/n_{sub}\;$$

When considering transmittance, only the zeroth diffraction order can be seen if *P* < |*mλ*_0_/*n*_sub_|. However, to make the higher-order diffracted light transmit into the substrate, the condition must be *P* > |*mλ*_0_/*n*_sub_|.

The condition of Wood’s anomaly is given by Equation (3):

(3) Re(kspp)= k0sin(αinc) ±mD. 

where Re(*k_spp_*) is the real value of the surface plasmon polariton wavenumber, and *D* = 2π/*P* is the grating wavenumber [31,32,33,34,35]. By simplifying, Wood’s equation can be written as

(4) $$\;P=\;m\lambda_{0}/{\left[\left(\epsilon_{sub}\epsilon_{Au}\right)/(\epsilon_{sub}\;+\;\epsilon_{Au})\right]}^{1/2}\;$$

where *ϵ_sub_* and *ϵ_Au_* are the permittivities of the dielectric and the metal, respectively.

## 3. Results

**Figure 2:**
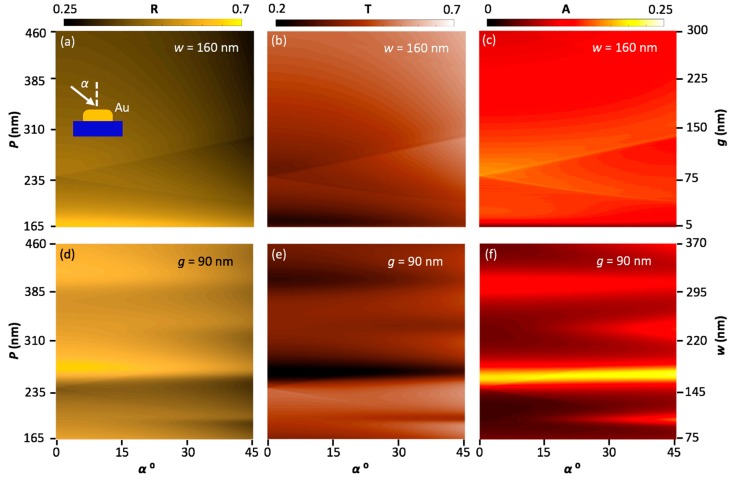
Color maps of reflection (R), transmission (T) and absorption (A) as a function of angle of incidence (*α*). In (**a**–**c**), the period was changed by varying the nano-slit width (*g*), and in (**d**–**f**), by varying the wire width (*w*). In (**a**–**c**), *w* and *t* are fixed at 160 and 15 nm, respectively, while in (**d**–**f**), *g* and *t* are fixed at 90 and 15 nm, respectively. Also, *g*, *w*, and *α* were swept from 5 to 300 nm, 75 to 370 nm, and 0° to 45° in steps of 1 nm, 1 nm, and 1°, respectively.

Models used in previous works [36,37,38,39] were updated and used to conduct this research. As mentioned, one period was modeled in the calculations, but periodic boundary conditions were applied in the x-direction to represent an infinite array. In this section, we focus on the effect of *P* and *α* on optical enhancement. Figure 2 shows reflection, transmission, and absorption as a function of *P* and *α*. Since *P* is a function of *w* and *g*, we studied the impact of changing *P* by individually varying both *w* and *g*. Figure 2a–c are results with a constant width, and Figure 2d–f are results with a constant gap size. Figure 2a represents the reflection as a function of *g* (or *P*) and *α*, which were swept from 5 to 300 (165 to 460) nm and 0° to 45°, in increments of 1 nm and 1°, respectively. The thickness, *t*, was fixed at 15 nm, and the wire width, *w*, was fixed at 160 nm. This width has a strong plasmon resonance at 875 nm. This produced the optimal enhancement, which represented the maximum ratio of the enhanced electric field in the substrate, |*E*_local_|, to the incident electric field, |*E*_0_|, all squared [16,37]. When the slit was very small, the structure was expected to produce high reflection because the filling factor, the ratio between *P* and *g*, was very high. By increasing the gap, *g*, the filling factor, and therefore the reflection, decreased as more light was able to transmit through the grating.

Figure 2d, where *P* is a function of *w*, shows a different trend in the reflection color plot from that in Figure 2a. This change is due to the different modes generated by surface plasmon polaritons. In Figure 2a, Rayleigh’s anomaly is dominant because *w* is limited. However, in Figure 2d Rayleigh’s anomaly is very weak, but surface plasmon polaritons are dominant because the wire width is increasing, which can generate multiple surface waves, as we describe later in this work. Figure 2e,f have a significant variation in the absorption and transmission spectra at *α* = 0°, *P* = 258, *w* = 168, and *g* = 90 nm. This change is attributed to surface plasmon polaritons. It is known that reflection is a function of *α* [40], and a significant change in the optical response can take place when the wave’s angle of incidence varies from normal to oblique. At certain values of *P* (*g*) in Figure 2a–c, the optical response changes when *α* changes. When the light is incident with a certain oblique angle, two distinct Rayleigh’s anomalies appear in the spectrum at every single diffraction order, as determined by Equation (1). This trend was very weak in the case of sweeping *w*, as in Figure 2d–f, because of the dominance of surface plasmon polaritons, as mentioned above.

**Figure 3:**
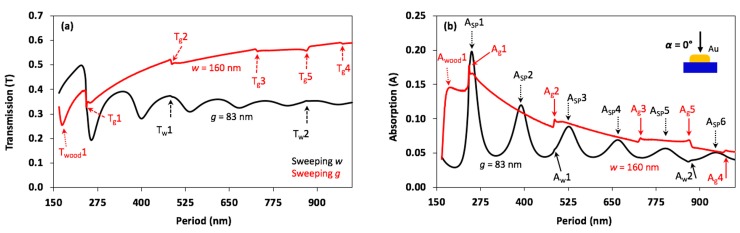
Transmission (T) and absorption (A) plots as a function of structure period at normal incidence. The red curves show the result of varying the period as a function of slit width (*g*) with *w* = 160 nm, and the black curves show results for varying the period as a function of wire width (*w*) with *g* = 83 nm. In (**a**), the transmission spectrum is plotted, with Rayleigh’s anomaly positions emphasized via dashed black and red arrows. These anomalies are located at *P* = 243, 487, 731, 868, and 961 nm, labeled as T_g_1/A_g_1, T_g_2/A_g_2, T_g_3/A_g_3, T_g_4/A_g_4, and T_g_5/A_g_5, respectively. T_w_1/A_w_1 and T_w_2/A_w_2 can be clearly seen due to the effect of surface plasmon polaritons. In (**b**), the absorption spectrum, Raleigh-Wood anomalies and surface plasmon polaritons are shown. Solid red and black arrows correspond to the positions of dashed red and black arrows in (**a**). Black dotted arrows in (**b**) refer to surface plasmon resonances at peaks A_sp_1, A_sp_2, A_sp_3, A_sp_4, A_sp_5, and A_sp_6. One diffuse peak, T_wood_1 or A_wood_1, which belongs to Wood’s anomaly, is seen in the absorption spectrum in the case of sweeping *g*.

Transmission and absorption were calculated and are plotted as a function of *w* (black lines) and *g* (red lines) in Figure 3. In previous work [36], the first peak in the spectrum was located at *w* = 160 nm; this wire width was chosen for the current study. At normal incidence, *g* was swept from 5 to 840 nm (*P* = 165 to 1000 nm). Depending on the absorption spectrum when sweeping *g*, the optimal slit (*g* = 83 nm) was chosen and held constant for the sweep of *w*. *w* was then swept from 82 to 917 nm (*P* = 165 to 1000 nm). Sharp dips and peaks in the transmission and the absorption spectra, which can be attributed to the Rayleigh anomaly, are visible in Figure 3a,b. According to Equation (2), the calculated resonant values of *P* are 242, 484, 726, and 968 nm when *m* = 1, 2, 3, and 4, respectively for the GaAs-Au interface, and at 875 nm when *m* = 1 for the air-Au interface. The resonant *P* values obtained by the computation are 243, 486, 731, and 975 nm, labeled T_g_1/A_g_1, T_g_2/A_g_2, T_g_3/A_g_3, and T_g_4/A_g_4, when *m* = 1, 2, 3, and 4, respectively, for the GaAs–Au interface, and at 868 nm, labeled T_g_5/A_g_5, when *m* = 1 for the air–Au interface. In addition, only one single diffuse resonant peak, belonging to Wood’s anomaly, was found at *P* = 190 nm, labeled T_wood_1/A_wood_1, which is very close to the calculated resonant *p* value of 187 nm according to Equation (4).

The analytical and the computational period values for Rayleigh-Wood anomalies were well matched. Rayleigh’s anomalies were located in the same positions in both cases, sweeping *g* and *w*, because they had the same periods. Again, since these anomalies were very weak compared with surface plasmon polariton effects in the case of sweeping *w*, only some of the anomaly peaks, such as T_w_1/A_w_1 and T_w_2/A_w_2, could be noticed. In the absorption spectrum shown in Figure 3b, increasing *w* generates several peaks at A_sp_1-6, which belong to surface plasmon resonances, and no Wood’s anomaly is observable. The plasmonic resonance peaks become wider and decrease in intensity, which means the enhanced electric field becomes weaker as *w* increases.

## 4. Electric Field Distributions

**Figure 4:**
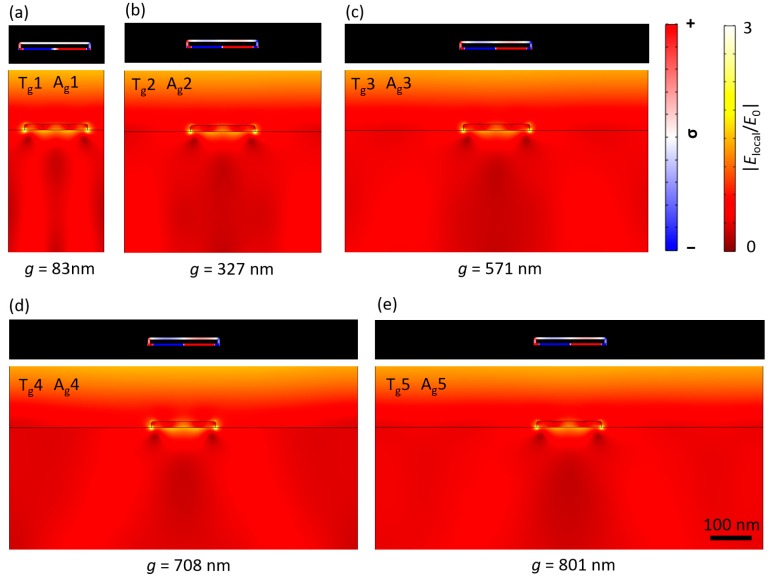
The electric field and charge distributions at Rayleigh’s anomaly as shown in Figure 3 for sweeping (*g*). (**a**) T_g_1 or A_g_1 at *P* = 243 nm, (*w* = 160 nm and *g* = 83 nm); (**b**) T_g_2 or A_g_2 at *P* = 487 nm, (*w* = 160 nm and *g* = 327 nm); (**c**) T_g_3 or A_g_3 at *P* = 731 nm (*w* = 160 nm and *g* = 571 nm); (**d**) T_g_4 or A_g_4 at *P* = 868 nm, (*w* = 160 nm and *g* = 708 nm); and (**e**) T_g_5 or A_g_5 at *P* = 961 nm (*w* = 160 nm and *g* = 801 nm).

This section focuses on optical enhancement, shown in plots of electric field and charge distribution generated by Rayleigh-Wood anomalies and surface plasmon polaritons, as shown in Figure 3b. Optical enhancement is defined as the absolute value of the ratio between the local electric field, |*E*_local_|, and the incident electric field, |*E*_0_|, all squared. However, only the enhanced electric field, |*E*_local_/*E*_0_|, is considered in this paper. The normalized surface charge density (σ) is used to illustrate the charge distributions. Figure 4 shows color maps of the electric field and charge distributions at Rayleigh’s anomaly for values of *g* corresponding to peak positions in Figure 3.

The dips in the transmission spectrum (T_g_1, T_g_2, T_g_3, T_g_4, and T_g_5) and the peaks in the absorption spectrum (A_g_1, A_g_2, A_g_3, A_g_4, and A_g_5) were located in the same positions (Figure 3). In the case of sweeping *g*, all generated resonance modes on the top and bottom surfaces of the nanowires were the same, and they radiated in all directions. One small difference is shown in Figure 4d when *P* = 868 nm, which is very close to the incident wavelength *λ*_0_ = 875 nm. This difference was due to the occurrence of the first diffraction order, *m* = 1, for the air–Au interface, which increased the charge distribution on the top surface of the nanowire. In addition, higher electric field values could be observed in the substrate and superstrate at this period; this could be attributed to the unique resonance mode resulting in stronger constructive interference.

**Figure 5:**
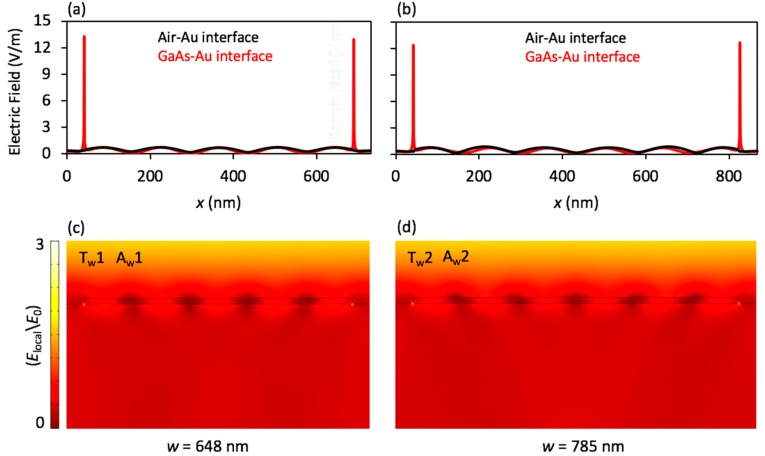
(**a**,**b**) Electric field distributions on the top and the bottom interfaces of the nanostructure and (**c**,**d**) for the entire model space at Rayleigh’s anomalies as shown in Figure 3, for sweeping *w*. In (**a**) and (**c**), T_w_1 or A_w_1 is located at *P* = 731 nm, (*w* = 648 nm and *g* = 83 nm), and in (**b**) and (**d**), T_w_2 or A_w_2 is located at *P* = 868 nm, (*w* = 785 nm and *g* = 83 nm).

In the case of sweeping *w*, the electric field distribution is shown in Figure 5 for the Rayleigh’s anomaly periods T_w_1/A_w_1, and T_w_2/A_w_2, as labeled in Figure 3. As shown in Figure 5a–c, the modes at the top and bottom interfaces were mostly identical, but at the edges of the bottom structure interface, the electric fields were stronger due to both the concentration of charge density at the sharp edges and the high relative permittivity of the GaAs. Since the nature of the dipoles generated at the wire edges were stronger than those in the middle, every individual dipole radiated as an individual source and constructively and deconstructively interfered within the substrate.

**Figure 6:**
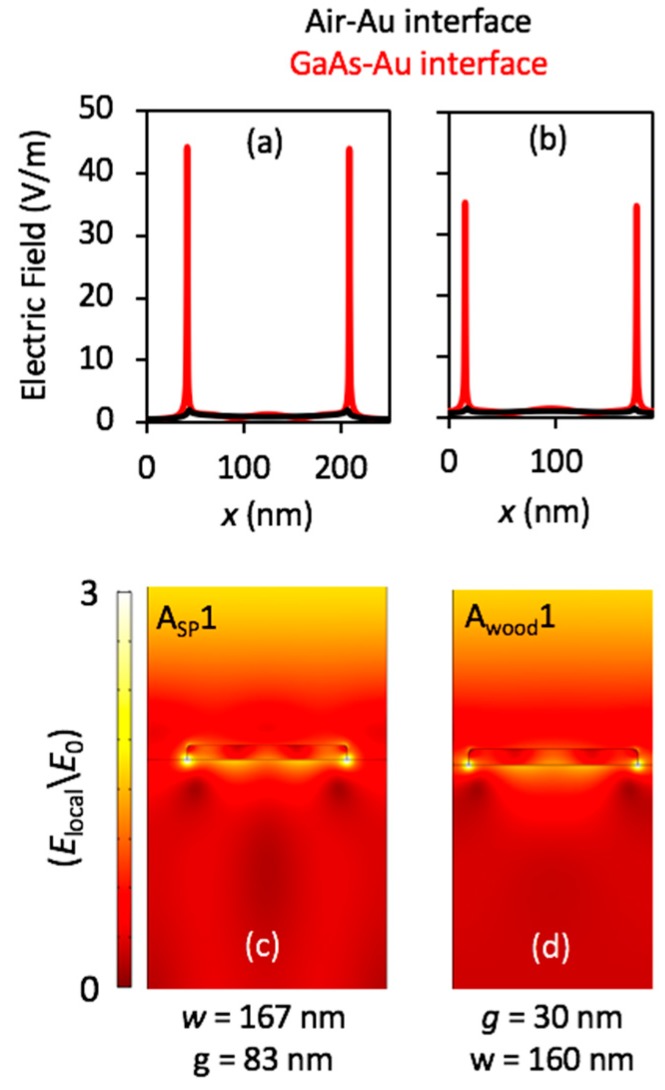
(**a**) and (**c**) A_sp_1 and (**b**) and (**d**) A_wood_1 peaks as shown in Figure 3. (**a**,b) Electric field distributions on the top and the bottom interfaces of the nanostructure; and (**c**,**d**) over the entire model space. (**a**) and (**c**) are when *P* = 250 nm (*w* = 167 nm and *g* = 83 nm), and (**b**) and (**d**) when *P* = 190 nm (*w* = 160 nm and *g* = 30 nm).

The electric field distributions at A_sp_1 and A_wood_1 peaks for the top and bottom interfaces, and for the entire model space are shown in Figure 6.

The results indicate that the electric field was enhanced 48 times in the substrate at the first surface plasmon resonance peak, which may be useful for increasing the current density in photodetector applications, for example. The enhancement, however, reached up to 35 times in the substrate for the Wood’s anomaly, as shown in Figure 6a,b. Various types of modes were generated at these peaks, and each type interacted uniquely with the incident light in the substrate, as shown in Figure 6c,d. This enhancement could be used to improve the performance of metal-semiconductor-metal photodetectors and solar cells.

## 5. Conclusions

We numerically studied the impact of grating period, wire and nano-slit widths, and incident angle of light wave on optical enhancement. Transmission and absorption were calculated and plotted as a function of grating period at normal incidence. Sudden changes in the spectrum, Rayleigh-Wood anomalies, and surface plasmon peaks occurred due to the resonant grating periods. Sharp peaks and dips that belong to these anomalies appeared at *P* = 243, 486, 731, and 975 nm, when *m* = 1, 2, 3, and 4 for the GaAs–Au interface, and at 868 nm when *m* = 1 for the air–Au interface. Wood’s anomaly, at *P* = 190 nm, was observed when the nano-slit was swept. Although these anomalies may seem weak—along with current density—they can make a significant improvement to the overall photodetector current [36]. Electric field and charge distributions and surface plasmon resonances were plotted at these anomalies. The peaks from sweeping the nano-slit were sharper and taller than those produced from sweeping wire width. The calculated and simulated Rayleigh-Wood periods were well matched. These results will be valuable for designing plasmonic grating structures for optimal light enhancement in photodectors and other optical applications.
